# Accurate and Low-Complexity Auto-Fingerprinting for Enhanced Reliability of Indoor Localization Systems

**DOI:** 10.3390/s21165346

**Published:** 2021-08-08

**Authors:** Elias Hatem, Sergio Fortes, Elizabeth Colin, Sara Abou-Chakra, Jean-Marc Laheurte, Bachar El-Hassan

**Affiliations:** 1 School of Engineering, EFREI Paris, 94800 Villejuif, France; elizabeth.colin@efrei.fr; 2 Faculty of Technology, Lebanese University, Aabey 1501, Lebanon; sabouchakra@ul.edu.lb; 3 Faculty of Engineering, Lebanese University, Tripoli 1300, Lebanon; bachar_elhassan@ul.edu.lb; 4 Electronics, Communication Systems and Microsystems Laboratory (ESYCOM), Université Gustave Eiffel, 77420 Champs-sur-Marne, France; jean-marc.laheurte@univ-eiffel.fr; 5 Instituto de Telecomunicación (TELMA), Universidad de Málaga, CEI Andalucía TECH, E.T.S. Ingeniería de Telecomunicación, Bulevar Louis Pasteur 35, 29010 Málaga, Spain; sfr@ic.uma.es

**Keywords:** two-wheeled robot, Received Signal Strength, auto-fingerprinting, RFID tag, position error, localization

## Abstract

Indoor localization is one of the most important topics in wireless navigation systems. The large number of applications that rely on indoor positioning makes advancements in this field important. Fingerprinting is a popular technique that is widely adopted and induces many important localization approaches. Recently, fingerprinting based on mobile robots has received increasing attention. This work focuses on presenting a simple, cost-effective and accurate auto-fingerprinting method for an indoor localization system based on Radio Frequency Identification (RFID) technology and using a two-wheeled robot. With this objective, an assessment of the robot’s navigation is performed in order to investigate its displacement errors and elaborate the required corrections. The latter are integrated in our proposed localization system, which is divided into two stages. From there, the auto-fingerprinting method is implemented while modeling the tag-reader link by the Dual One Slope with Second Order propagation Model (DOSSOM) for environmental calibration, within the offline stage. During the online stage, the robot’s position is estimated by applying DOSSOM followed by multilateration. Experimental localization results show that the proposed method provides a positioning error of 1.22 m at the cumulative distribution function of 90%, while operating with only four RFID active tags and an architecture with reduced complexity.

## 1. Introduction

In modern life, applications of mobile robots have expanded their scope to autonomous security guards, guidance for elderly people and a variety of industrial automations. These automatic systems need to know the robot’s position in order to follow its navigation and perform actions in the considered environment.

Global Navigation Satellite System (GNSS) solutions, such as Global Positioning Systems (GPS), are the most extensively used architectures to provide positioning in outdoor environments. The low-cost of localization systems and their accuracy and the lack of any pre-requirement or measurement to be performed before their use allow them to support any outdoor mobile navigation application, including implementations for pedestrians, cars, robots, flying drones, etc. [1,2].

However, the effect of obstacles and Non-Line-Of-Sight (NLOS) propagation [3] make GNSS essentially unavailable or very inaccurate in indoor scenarios [4]. This implies a huge barrier for the implementation of many applications of positioning, logistics [5], games and augmented reality applications [6,7] and even the management of cellular networks [8,9,10,11] indoors.

To overcome this issue, specific localization methods are needed. While multiple techniques and technologies have been proposed, the approach based on fingerprinting via the Received Signal Strength (RSS) may be the most common [12], where the radio technology may vary between Radio Frequency Identification (RFID) [13], Wireless Fidelity (WiFi) [14], Bluetooth [15] and cellular [16] methods.

Indoor localization based on fingerprinting relies on two stages: the training stage, where a large set of training data is acquired to create the radio map and model the signal environment, and the estimation stage, where the mobile position is estimated based on the training data and a new observation of the environment. Therefore, most fingerprint positioning methods need to collect a large amount of data, and the positioning investigation requires manpower and is very time-consuming; all this complicates the localization method. Thus, robots may be adopted as a dedicated surveyor to fingerprint the environment autonomously [17,18,19].

In this regard, the present work proposes an auto-fingerprinting method for localization using RFID, featuring low training complexity as well as high estimation accuracy. The proposed method is implemented and evaluated in a practical indoor case study. The localization test is divided into offline and online stages: the offline stage involves an auto-training phase to collect RSS values for the environment calibration obtained by applying the Dual One Slope with Second Order propagation Model (DOSSOM) [20], while in the online stage, the robot’s position is estimated by again using the propagation model DOSSOM, followed by the multilateration technique. The contributions of this paper are three-fold: first, only one RFID tag is needed for the training phase, while a high number of deployed tags is used for similar fingerprint scenarios [21,22]. Second, our method makes the fingerprinting of the indoor environment cheap and exhaustive and reduces the time-consumption to reasonable levels. Finally, the auto-fingerprint method also enables reliable data capture for the localization stage.

The rest of the paper is organized as follows: Section 2 introduces the key related works in the domains of indoor localization and robot utilization for fingerprinting. In Section 3, the suggested robot displacement study and the description of the auto-fingerprinting method are validated by localization results. Finally, the last section concludes with the findings of this work.

## 2. Related Works

Several technologies are used to implement indoor localization systems. Researchers have proposed different approaches to realize accurate fingerprint techniques in various indoor environments. These approaches have their own advantages and limitations. Increasing numbers of studies focus on an automated and accurate fingerprinting method for indoor positioning and navigation systems [23,24,25,26,27,28,29,30,31,32,33,34,35,36,37,38,39,40,41,42]. This section summarizes the main localization methods realized by users or by robots and presents a discussion of these methods. While multiple radio technologies are used, such as WiFi, cellular, RFID and Bluetooth, fingerprinting based on RSS and conventional positioning techniques are applicable for all of these. Therefore, the bibliography covers works on multiple technologies, focusing on their general positioning mechanisms and procedures, thus allowing us to analyze those involving robot-assistance in their working process.

### 2.1. Survey on User Fingerprinting and Positioning

The main references in the field of RSS-based fingerprinting are elaborated in [19]. Particularly, in [23], a novel crowd sourcing method is proposed for both radio map construction and updates to the map in order to reduce the site surveying time. Here, a fusion of RFID, Pedestrian Dead Reckoning (PDR) and Magnetic Matching (MM) technologies for indoor localization is presented. It is noticeable that the obtained positioning error is equal to 2.4 m on average. In addition, in [24], an Unsupervised Indoor Localization (UILoc) system is presented that combines smartphone sensors, iBeacons and WiFi to improve the system reliability and the location accuracy without any labor cost. The UILoc system was implemented in a typical 3000 m^2^ office building, reaching an average localization error of 1.11 m. However, the presented RSS-based localization system faces the challenge of huge computational complexity, an increase of the location prediction time and inconsistent performance due to the fusion of three technologies.

Looking at Deep Learning (DL) approaches, in [25], the transformation of WiFi signatures into images and the creation of a scalable fingerprinting framework based on Convolutional Neural Networks (CNNs) with five layers are proposed. This approach is performed in three different indoor paths. Experimental results show that an average positioning error of under 2 m is achieved. In the same context, the work in [26] develops a CNN model for WiFi-based localization. Its analysis is conducted over the entire floor of a building. The proposed system is divided into offline and online stages. A four-layer CNN structure is trained to build fingerprints during the offline stage; the target position is then estimated online. Compared to five different CNN applications, the proposed model features improvement regarding the instability and variability of the Received Signal Strength Indicators (RSSIs) for WiFi signals and an average location error of 1.44 m. Furthermore, in [27], a localization system is presented that employs a wireless fingerprint based on CNN. The DL method was used to obtain the characteristics of the fingerprint during the offline stage and predict the indoor location within the online stage. This method was deployed in a laboratory of the Engineering Faculty building. Finally, the empirical results showed that the CNN significantly improved the positioning system’s accuracy compared to the performances of the K-Nearest Neighbor (KNN) and Support Vector Machine (SVM) approaches. The work in [28] presents a fault-tolerant indoor localization system based on Recurrent Neural Networks (RNNs). Different types of RNN architectures were evaluated by correctly classifying the target’s location in an entire floor. It was shown that the use of 50 Gate Recurrent Units (GRU) with 5 routers was the suitable architecture, reaching the highest degree of hit-rate accuracy at 87%. Hence, although providing good results, DL approaches for RSS-based indoor positioning system have a major shortcoming: the need to have a large amount of labeled RSS input data and convolutional filters to train the system, which are difficult to acquire in real deployments [24,25,26,27].

Conversely, the work in [29] proposes a more straightforward template matching algorithm for a Bluetooth Low Energy (BLE) fingerprint indoor localization system. For each Access Point (AP), the indoor environment is divided into four quadrants; the template parameters are chosen based on the differences between RSSIs at the center points and their eight neighbors in the different quadrants, respectively. The proposed algorithm was tested in a 64 m^2^ reading room, deploying 4 APs and 7 Reference Points (RPs). The achieved location accuracy was 1.1 m at the 80th error percentile. However, an increase in the indoor environment space requires more human effort for RSSI collection and more data in the radio map database, and the number of RPs with an abnormal RSS will increase. Hence, the use of advanced filters is recommended; however, this increases the system’s cost and complexity.

Going beyond pure positioning accuracy, in [30], the authors suggest a combination of battery-saving techniques with a localization system based on WiFi fingerprinting. These novel techniques adapt the scanning frequency to the user’s physical activity to save energy. They take the data set and send WiFi scans, without affecting the user’s device. Using 460 APs, tests were carried out on the first and ground floors of the Escuela Técnica Superior de Ingeniería Informática, in Seville, Spain. The average localization error obtained was 4.51 m. With these techniques, RSS signals collected from the large number of APs are entirely processed in the device before being uploaded to the server. In the same context of energy saving, in [31], an adaptive wireless indoor positioning system is developed based on the transmitter power control algorithm. This localization system makes an adjustment to the positioning accuracy at the assumed level of energy. Several test scenarios were performed to verify the system’s performance. The highest average localization accuracy of 33 cm was reached with the minimum energy savings. In both studies [29,30], the system response time and the risk of not having a reliable user position in emergency conditions are elevated.

In [32], the authors investigate WiFi-fingerprint-based localization in highly dynamic indoor environments. In fact, spatio-temporal variation is one of the intractable problems of indoor settings. To overcome these effects, an Expectation–Maximization (EM)-based filter is proposed to train the dataset with a binary hidden variable to identify and remove abnormal RSS values. A simultaneous AP selection and localization approach is proposed for optimal matching in the test phase by employing a Bayesian framework. Measurements were realized over the whole floor of a building of 1200 m^2^, arranging 30 APs and 499 RPs. Experiments demonstrated that the proposed scheme had a considerably low position error of 4 m at the 90th percentile. In addition, in [33], a new positioning method with Bayesian tracking is proposed that consists of three stages. In the first, a fingerprint model is built for two different environments: a 162 m^2^ empty room and 58.8 m^2^ office including people as well as furniture. In the second stage, the generated model is used with the maximum likelihood criterion to obtain a preliminary position estimate based only on the fingerprint generated at the receiver’s position. Finally, in the third stage, a maximum likelihood Kalman filter is used to combine the preliminary approximation with the dynamical model of the receiver’s motion to obtain the final estimation. Using two APs in each environment, experiments show that the localization method is accurate in both indoor environments, achieving an average positioning error of 1.09 and 1.45 m in the room and the office, respectively. In both studies [31,32], the positioning stage requires a small number of training data acquisitions to be computed. However, if the assumption of independence among the data set does not hold, the performance of the filters would be very limited.

Nevertheless, all fingerprinting techniques still have some common and important challenges such as the instability of RSSI measurements affected by the multipath effects at a given location (due to the environmental factors), human error and the high manpower/time costs for data collection. These drawbacks lead to the need for better approaches, where benefiting from robot support in the fingerprinting and positioning procedures may be key.

### 2.2. Survey on Robot Fingerprinting and Positioning

In the recent past, mobile robot indoor positioning has been the focus of multiple activities that have aimed to optimize time and manpower significantly compared to manual position determination [34,35,36,37,38,39,40,41,42]. The work in [34] studies two parameters that might affect measurement quality: the WiFi Access Point (AP) antenna height and the WiFi AP–Receiver distance separation. They use statistical analysis—i.e., an Analysis of Variance (ANOVA)—to check whether these parameters affect the positioning system’s accuracy. The assessment was conducted on the second floor of the Center for Human–Robot Symbiosis Research, Toyohashi University of Technology, and showed an accuracy of 1.8 m at the 90th error percentile. This approach is limited to only two parameters, while many other factors can also affect the indoor location accuracy.

In [35], the authors consider automatic data collection for indoor localization purposes using the Simultaneous Localization and Mapping (SLAM) algorithm. RSS values were acquired on the third floor of a university building via a robot equipped with an Android phone, odometer and gyroscope. The proposed system comprises auto-calibration and positioning stages. The measurement accuracy with the robot-based localization, is improved by 10% in comparison to manual localization. Despite the improvement achieved by the proposed RSS-positioning system, some issues were not addressed, such as the configuration of the training points for successive data acquisitions and the long RSS acquisition duration.

Beyond this, the work in [36] improves the fingerprinting data collection stage and the positioning accuracy. The authors propose the idea of time synchronization while collecting the RSS values and locating the mobile robot. This method is called the Tensor Nuclear Norm (TNN), where the accuracy of the proposed method reaches 2 m. This positioning system does not take into account the robot’s displacement error that may accumulate in large indoor environments.

In the same context, the work in [37] develops an adaptive wireless positioning system based on an autonomous database updated by an Adaptive Signal Mode Fingerprinting algorithm (ASMF) for complex indoor environments. The proposed system was tested in a 147 m^2^ laboratory. According to the experimental results, the presented localization algorithm reached a higher accuracy compared to KNN in different indoor scenarios. It showed an average positioning error of 84 cm but used 531 RPs. Moreover, this approach has some unavoidable limitations such as the influence of moving obstacles and the local optimum problem in optimizing the shadowing model process. In addition, in [38], an automated signal mapping robot is proposed called RobotMapper. The approach focuses on mitigating the required time and human resources by automating and simplifying the repetitive characteristic of the learning phase in fingerprinting. This system is implemented and evaluated in a 47 m^2^ laboratory, achieving a mean localization error of 2.21 m. With this RSS-based localization system, the robot traverses the environment and visits each RP by self-control with the help of a given RP database. In [39], an automated model to construct and optimize fingerprint databases is presented. This model uses an initial radio map based on a theoretical path loss model, unlabeled training data, a self-calibration method and a route mapping filter. Experiments were carried out in 1100 m^2^ offices and reached 5 m of location accuracy at the 90th percentile of error. They did not take into consideration the high time requirements of their measurement campaigns, device calibration or additional inertial measurement units.

Furthermore, the work in [40] implements a new DL technique based on RSS values for fingerprinting. This generates augmented RSS data that mimic the original acquisitions in order to generate the RSS data set. This can be integrated with fingerprinting to enlarge the training dataset. The mean location errors achieved were 1.45 and 1.60 m in 1664 m^2^ simulated and real laboratories, respectively. This technique presents some challenges with WiFi localization regarding the network management, which changes the Radio map of the environment and increases the error. In subsequent research, in [41], a Soft Range Limited K-Nearest Neighbors (SRL-KNN) localization fingerprinting algorithm was developed. A remote three-wheeled robot was used to test the proposed localization algorithm in a 336 m^2^ part of the Engineering Office Wing (EOW), University of Victoria. The proposed algorithm can effectively address some challenges. Experimental results proved that the proposed method reached an accuracy 1.1 m at the 90th error percentile while using 365 RPs. This obtained localization accuracy is due to the large number of RPs, which increases the complexity of the system computation.

Recently, in [42], a new dual-frequency Phase Difference of Arrival (PDoA)-based indoor localization system using a mobile robot was proposed. The experiment was carried out in a simulated two-dimensional 25 m^2^ area. The distance between two adjacent passive UHF tags was 0.5 m. To mitigate the tracking problem using the new Kalman Filter algorithm, the odometry information, obtained from wheel encoders, was fused with the RFID localization results. The RFID localization errors achieved were 0.148 and 0.144 m, while deploying 10 RFID tags over the straight line and the circle trajectory, respectively. Despite the high localization accuracy achieved, the proposed system was not evaluated in a real-world scenario, where the filter performance is usually limited if the independence of the data set does not hold.

This analysis of the bibliography indicates that the robot calibration, RSS instability and RSS collecting time are still challenges that require further developments in this area. In this field, the present work provides a simple and accurate auto-fingerprinting method for an indoor robot positioning system based on RFID technology. The robot’s calibration aims to mitigate the robot displacement error and improve the radio map’s reliability. Regarding the system complexity, only one RFID tag is used for the environment calibration and four are used for position estimation, hence reducing the system’s complexity and cost. In order to assess our approach, Section 4 compares our method with the systems presented in [34,35,36,37,38,39,40,41,42].

## 3. Experimental Setup and Localization Processing Details

Most mobile robots introduce systematic errors caused by imperfections in the design and mechanical implementation [19]. Therefore, the calibration of robots is a key process to achieve proper results in the odometry-based navigation of any moving system.

In this context, we investigate the robot displacement issue with the aim of improving the reliability of auto-fingerprinting as well as the localization accuracy. We start with an overview of the robot’s systematic errors and the method of calibration used typically to keep it on the considered trajectory and collect the RSS acquisitions accurately in both offline and online stages of our auto-fingerprinting approach.

### 3.1. Robot’s Displacement Evaluation

When aiming to improve auto-fingerprinting and typically to remain on track, the robot must be calibrated. Here, odometry is fundamental. Odometry is used in robotics to estimate a robot’s position relative to a starting location [43]; it handles motion data to estimate changes in position over time. Moreover, well-calibrated odometry is an essential phase for a mobile robot to have an accurate displacement over a long path; this can be achieved through different test scenarios.

**Figure 1:**
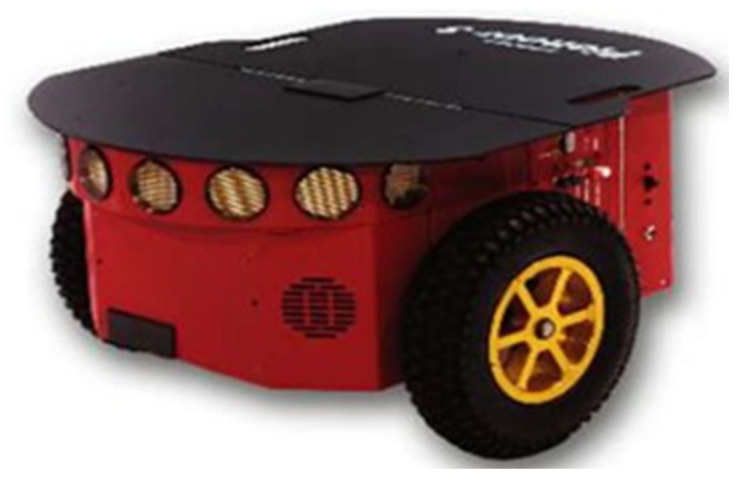
The Pioneer 3-DX mobile robot.

As a robot platform, the model Pioneer 3-DX [44], shown in Figure 1 was used in the experiment. The Pioneer 3-DX is a two-wheeled robot with dimensions of 45.5 × 38.1 cm. The Software Development Kit (SDK) provided by the manufacturer is used to control it in combination with the Advanced Robot Interface for Applications (ARIA), which is a C++ library for all mobile robot platforms, allowing access to all parameters, such as speed and heading.

For navigation, the two key factors are the robot’s deviation and stop estimation [45]. To guarantee accurate displacement, many experiments have been carried out on robot odometry errors, investigating factors such as moving in a straight line, the velocity of the wheels, the rotation of the wheels and square path calibrations.

#### 3.1.1. Straight Line Test

**Figure 2:**
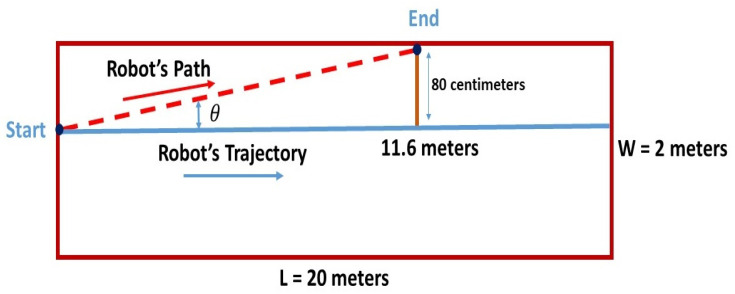
The diagram of the straight line test.

The projection of the wheelbase center is considered to be the robot’s location, as shown in Figure 2. The robot moves along a straight line of length L until it reaches the end position.

This test was done in a corridor whose dimensions were 22.5 × 2 m. The robot was placed at a distance of 80 cm from the left wall instead of the midline as the right wall of the corridor was not consistently straight. The expected robot’s trajectory was a straight line of 20 m. As shown in Table 1, three tests were carried out, and the deviation was calculated at each 1 m.

It can be seen that the robot deviated to the left and hit the wall at a distance of 11.6 m. This deviation can be neglected at the beginning; however, correction is required as the robot moves forward. Before evaluating the localization system performances, further investigations about the robot’s displacement are needed to correct its deflection. A straight trajectory may be obtained by changing the speed of the left wheel.

#### 3.1.2. Wheel Velocity Test

Next, a speed test for the wheels was applied in order to determine the origin of the robot’s drift away from the straight line. ARIA has some functions that make it possible to obtain the linear speed of each wheel. The test was done over a straight line of 5 m, displaying the speed of each wheel every second. The experiment was repeated three times. Angular velocities (rad/s) were converted into linear speeds (mm/s) using the equations expressed below:

(1) $$V_{l}=R\cdot W_{l}\;$$

and

(2) $$V_{r}=R\cdot W_{r}\;$$

where $V_{l}$ and $V_{r}$ are the linear speed of the left and right wheel, respectively; $W_{l}$ and $W_{r}$ are the angular velocity of the left and right wheels, respectively; and *R* is the wheel radius.

Knowing that the wheel radius was 92.5 mm [44], Table 2 represents the absolute difference between the speed of the right and left wheel.

Based on these tests, the difference between speeds was quite small, with a worst case of 0.04 mm/s. In short, the problem of the robot’s deviation was not due to the difference in the wheels’ velocity.

#### 3.1.3. *Wheels’ Rotation Test*

**Figure 3:**
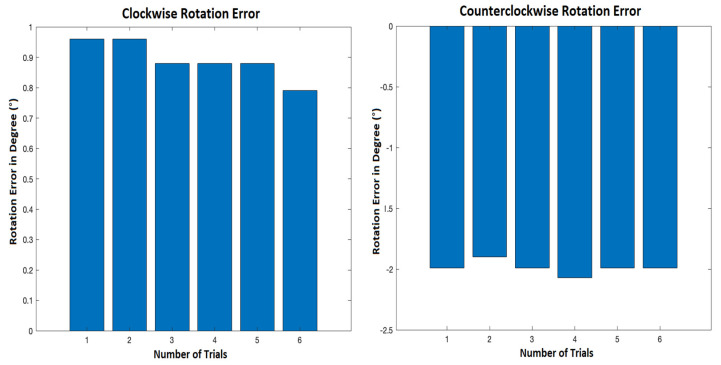
Clockwise and counterclockwise rotation error.

As the wheels speed is not behind the robot’s drift, wheels rotation test is necessary to analyze the wheels’ rotation stability. Thus, the robot was rotated about itself 360° at the same speed in two directions i.e., ClockWise (CW) and CounterClockWise (CCW). The difference in angular velocity between the two wheels was analyzed. Then, the angle deviations are measured in both directions. Figure 3 presents the rotation errors for the six trials.

Figure 3 shows that when the robot turned clockwise, it could almost turn 360°, whereas it rotated 359.1° on average. However, in a counterclockwise direction, the robot tended to rotate more than 360°, at around 362° on average. It can be seen that the wheel rotation was almost stable and cannot be considered to be the cause of the robot’s deviation. Hence, another test was finally conducted to show the robot’s performance in a complete cycle path.

#### 3.1.4. Square Path Test

**Figure 4:**
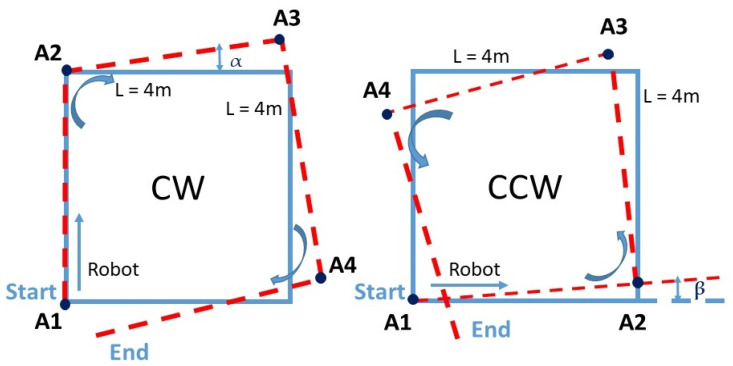
The diagram of a square path clockwise and counterclockwise.

For this test, the procedure defined as the University of Michigan Benchmark test (UMBmark) [46] was adopted as it was especially designed to uncover certain systematic errors. This method involves a set of test runs in which the robot is programmed to follow a 4 × 4 m square path, as shown in Figure 4. Due to systematic errors, after linear and turning movement, the robot had a position offset and could not return to the initial point.

For both CW and CCW scenarios followed by the mobile robot, offsets were studied from 10 trials. The average of each angle was calculated as shown in Table 3.

Considering the test results, it can be concluded that the robot tended to drift to the left. However, the deviation angle was relatively small compared to 90°. Over short distances (less than 1 m), and with the large size of the robot, which was equal to 45.5 cm, the deviation did not significantly affect the robot’s displacement accuracy; it corresponded to only 6.66% of the robot’s length.

Over a longer trajectory and referring to the straight-line test (Figure 2), the robot presented an angle deviation of $\tan^{-1}(\theta$) = 0.8/11.6 = 3.95° to the left. Thus, to have an accurate mapping coverage and stable motion, an auto-correction by a rotation of 3.95° clockwise needed to be applied.

### 3.2. Localization by Multilateration

Once the capabilities of the robot were known and the auto-correction was considered, the proposed auto-fingerprinting method was designed and implemented for the RFID-based localization system. To improve upon the time-consuming and labor-intensive user-based processes, the self-environment calibration required the construction of a signal strength map using the two-wheeled robot.

The system setup time, the human effort for configuration and the total cost of the equipment can be considered to be the cost of the positioning system. The system’s complexity is attributed to the hardware, software and operation factors. According to the literature [47], the cost and power consumption of using WiFi technology to realize an indoor positioning system is very high compared to other technologies. The RFID technology is viewed as a potential candidate as it requires relatively low configuration time and battery power as well as benefiting from easy control [13]. The choice of the robot and the RFID technology significantly affect the granularity, accuracy and cost of the proposed positioning solution. It is worth mentioning that employing a large number of APs and RPs can greatly improve the positioning accuracy, as in [29,30,32,37,38,41]. Furthermore, using effective data filtering and preprocessing operations is recommended, but it increases the system’s computing complexity. In our present work, we focused on implementing a simple localization system with a reduced number of deployed RFID tags.

**Figure 5:**
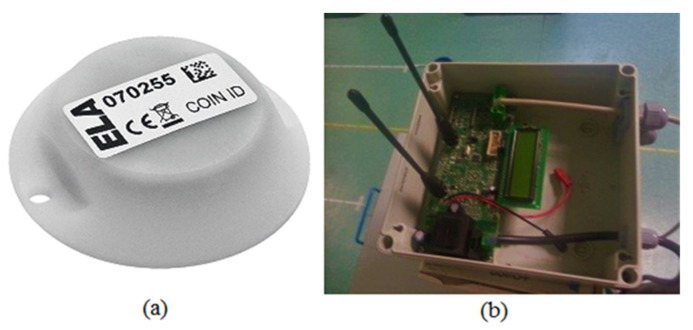
(**a**) Coin ID RFID tag and (**b**) UTP Diff 2 RFID reader.

In our case, the RFID system consisted of a reader, an active tag and digital signal processing algorithms applied on RSS values collected by the reader. Figure 5a shows the “Coin ID” tag from Ela-Innovation [48]. The operational frequency of the active UHF-RFID tag was 433 MHz. It could be fixed on the walls of the indoor environment. The RFID reader, shown in Figure 5b, was mounted over the robot. The RFID reader consumed on average 80 mA at 12 V, whereas RFID active tags are typically 3 V battery-powered.

**Figure 6:**
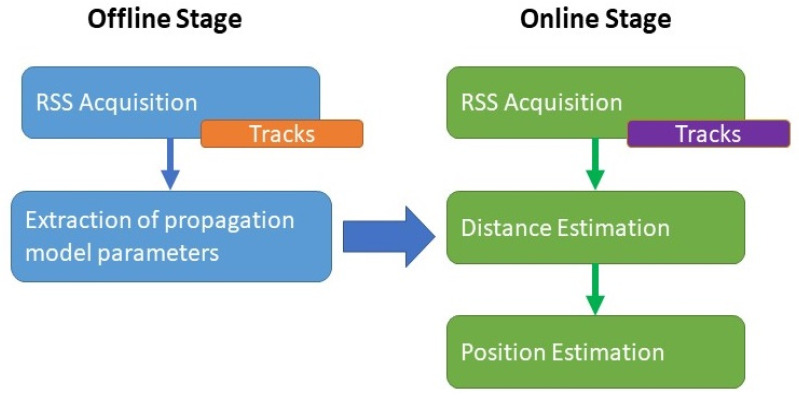
Block diagram of the offline and online stages.

The localization process was divided into two stages: offline and online, as presented in Figure 6. The offline stage represented the environment calibration, and the online stage was the positioning phase. During the offline stage, the environment was split into several “tracks” and RSS measurements were collected (“RSS acquisition”) at sampling locations over these tracks to build a radio map of the indoor environment. By applying the considered propagation model, the environment attenuation coefficient was determined to be represented by the “extraction of propagation model parameters” block.

After the correction of the robot’s displacement and the auto-fingerprinting, the robot’s position was then estimated, within the online stage, using the previously obtained propagation model followed by the multilateration technique based on the different RSSI values acquired over the trajectory.

#### 3.2.1. Offline Stage—RSS Acquisition

**Figure 7:**
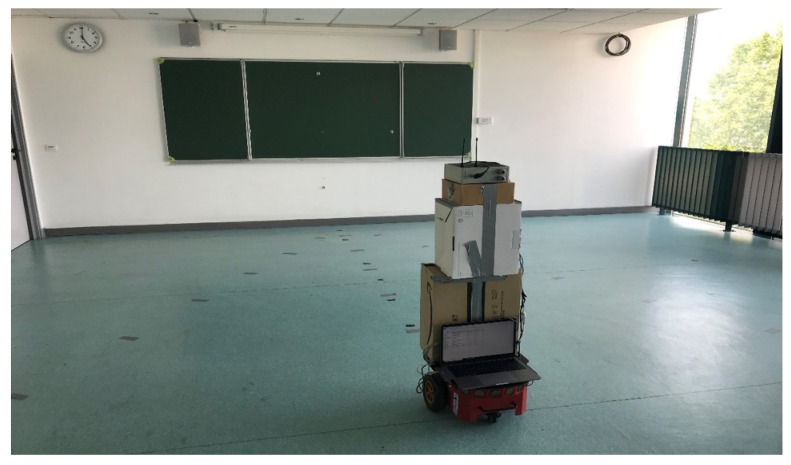
Experimental environment view.

**Figure 8:**
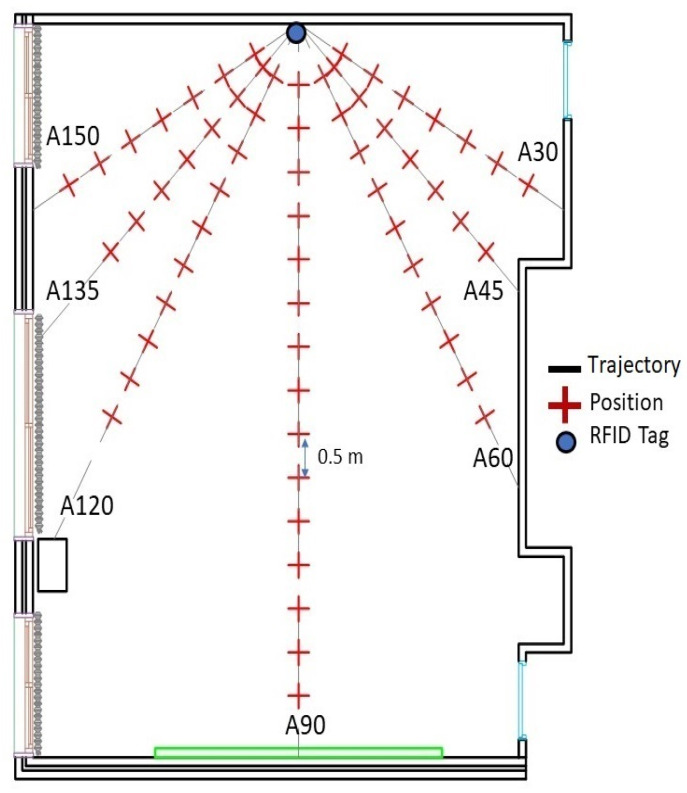
Auto-fingerprint map.

Fingerprinting was conducted in a classroom at EFREI-Paris with dimensions of 8.5 × 7.5 × 2.51 m. Figure 7 shows a picture of the scenario, whereas Figure 8 shows the layout. To characterize the behavior of the signal in the environment, seven paths, as shown in Figure 8, represent the robot trajectories. These radial paths, also called tracks, were used to conduct the fingerprinting, covering the entire indoor environment. This presented fingerprinting model aims to minimize the system cost by reducing the number of deployed RFID tags, as well as mitigating the robot’s displacement error.

In the auto-training phase, only one RFID active tag was used as an emitter. It was fixed on the center of the front wall. To cover the systematic errors of the robot, the proposed scenario consisted of moving the robot forward with a step equal to 50 cm and stopping to collect 200 RSS acquisitions at each position over the seven trajectories A30 to A150, as shown in Figure 8. These RSS acquisitions were combined via the averaging technique and converted into a power level in dBm at each position.

Based on the received power values gathered during the offline phase, the Dual One Slope with Second Order propagation model (DOSSOM), which was previously introduced in a previous publication by the authors of this work [20], was applied to feed the posterior online stage with accurate attenuation coefficients covering the considered indoor environment. The propagation model DOSSOM is expressed as follows:

(3) $$P\left(d\right)=\{\begin{array}{c} PL_{0}+10\cdot n_{T_{i}}\cdot log_{10}\left(d\right)+X_{T_{i}}\;\;d\leq 3\lambda \\ a\cdot \log_{10}{\left(d\right)}^{2}+b\cdot log_{10}\left(d\right)+c\;\;d>3\lambda \end{array}$$

where P(d) is the received power in dBm at distance *d* in meters, $PL_{0}$ is the free space path loss at the distance of 1 m, $n_{T_{i}}$ is the path loss exponent corresponding to the first part of the path, and $X_{T_{i}}$ is a lognormal variable for the received power error throughout the first part of each track modeled by the one-slope variation. *a*, *b* and *c* are the constant parameters of the second-order polynomial model. They are determined by solving a system of three unknowns that can be obtained by considering three pairs of particular values of *P* and *d*.

#### 3.2.2. Online Stage—Received Signal Strength Indicators (RSSIs) Acquisition

**Figure 9:**
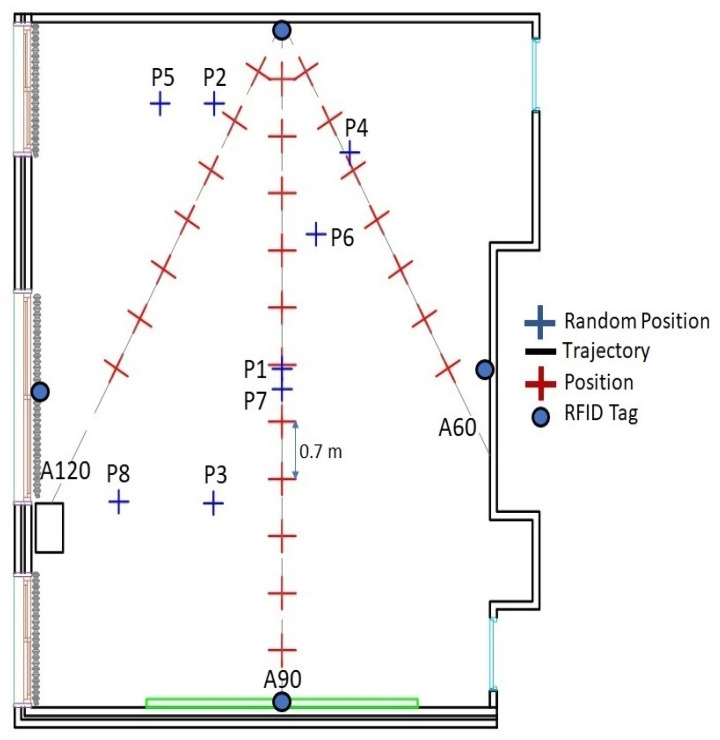
Two-dimensional configuration of the eight random positions.

To evaluate the effectiveness of the proposed localization system, RSS data at 32 locations were collected in the online phase. Twenty RSS samples were acquired at each position and combined by averaging. At each position, the average RSS value was converted into a power level in dBm. Twenty-four locations were uniformly distributed in the space with a distance of 0.7 m, as shown in Figure 9. In contrast, eight positions were chosen randomly to study the performance of the odometry and the accuracy of the automatic environment calibration method and analyze the position error.

**Figure 10:**
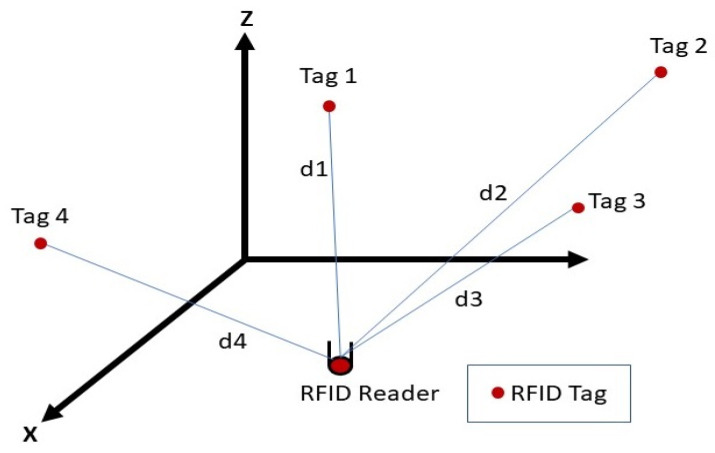
Multilateration technique using four independent tags.

To determine positions based on the multilateration technique, four independent tags are needed [49]. Tags were located at the center of each wall as shown in Figure 9. Knowing the power values and the attenuation coefficients determined in the offline phase and applying the DOSSOM model again (Equation (3)), the four distances between each tag and the reader, which was mounted over the robot, were estimated (Figure 10).

**Figure 11:**
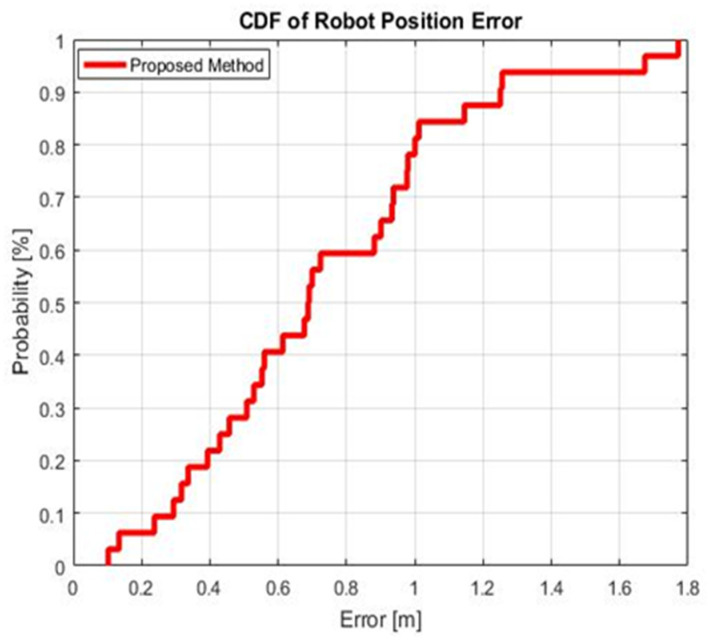
CDF for the position errors using the auto-fingerprinting technique.

Figure 11 presents the Cumulative Distribution Function (CDF) of the position error. Table 4 summarizes the results of different approaches for localization.

Looking at the values in Figure 11 and Table 4, the achieved position error at 90% of CDF was 1.22 m. The standard deviation of the localization is also highlighted to show the stability of the proposed system; it was only 0.42 m. Thus, the efficiency of the auto-fingerprinting method was validated for the localization purpose in an indoor environment, as its performance was comparable to those found in the state of the art.

Returning to related works on robot fingerprinting and positioning, Table 5 summarizes the obtained positioning accuracy of several applications based on auto-fingerprinting.

As summarized in Table 5, several systems have provided a number of auto-calibration and positioning solutions. However, they have focused generally on the location accuracy and neglected the complexity and cost of the system. The most successful robot-based localization algorithm seems to be the image processing proposed in [37,41]. The localization accuracy achieved a sub-metric order with a large number of RPs. The solutions in [34,35,36,38,39,40,41,42] are based on developing algorithms to improve the localization system performance without taking into consideration the robot’s displacement errors and the system’s complexity. Hence, the proposed system focused more on optimizing the robot’s displacement errors, which may accumulate over long paths indoor, while maintaining a low level of needed hardware. It can be observed that the localization via the proposed auto-fingerprinting method presented an accuracy of 1.22 m at 90% positioning error and a standard deviation of 0.42 m. According to these results, our proposed system is more accurate than the methods in [34,35,36,38,39,40] and simpler compared to the approaches adopted in [37,41,42] that employ complex algorithms, as well as filtering approaches, and that need a large number of deployed reference devices. In contrast, our localization system is simplified by treating the data set with a simple averaging RSS combing technique and deploying only four RFID active tags. It also presents better robot navigation stability than the methods in [34,37,38,40], with standard deviations of 0.49, 0.59, 1.28 and 1.83 m, respectively. The system’s positioning accuracy achieved at 90% of the CDF also demonstrates the effectiveness of the proposed approach and its superiority compared to the related works presented in [34,38,40,41], with values of 1.8, 3, 1.83 and 1.1 m at 90% of CDF, respectively.

## 4. Conclusions

In this paper, an efficient auto-fingerprinting method via a mobile robot was proposed. This was validated for indoor positioning using RFID technology. The auto-fingerprint method gathered measurements from a set of positions in a complex scenario in which the robot’s displacement accuracy was examined. On the other hand, the auto-correction based on odometry tests presented a reduction in the robot displacement uncertainty. Hence, the position accuracy was evaluated within the online stage and achieved an error of 1.22 m, with a cumulative density function at 90%, by implementing a cost-effective and reduced complexity architecture.

Considering the features of this auto-fingerprinting method in our experimental area, future work will focus on implementing it in other environments such as corridors, office floors or halls. It could also be proven that our localization system has the potential to be enhanced via different localization techniques to determine positions in navigation systems without a map.

## Figures and Tables

**Table 1:** Wheels’ Deviations over Straight Path.

Number of Tests	Test 1	Test 2	Test 3	Mean Deviation (cm)
Deviation/1 m [cm]	6.780	6.754	6.724	6.753

**Table 2:** Difference between wheel velocities.

Number of Tests	Test 1	Test 2	Test 3
$\left|V_{l}-V_{r}\right|$ (mm/s)	0.0419	0.0027	0.0297

**Table 3:** Robot angle deviation.

Angle	A1	A2	A3	A4
Clockwise (α)	90.1°	93.6°	92.4°	88.4°
Counterclockwise (β)	88.11°	88.66°	98.33°	81.11°

**Table 4:** Summary of localization results.

Algorithms	50%	90%	Min	Max	Std
Accuracy (m)	0.7	1.22	0.1	1.75	0.42

**Table 5:** Auto-fingerprinting systems.

System	Technique	Accuracy (m)
[34]	ANOVA	1.8
[35]	SLAM	1.5
[36]	TNN	2
[37]	ASMF	0.84
[38]	RobotMapper	2.21
[39]	DL	5
[40]	DL	1.6
[41]	SRL-KNN	1.1
[42]	PDoA + KF	0.14
Proposed System	Odometry	1.22

## Abbreviations

The following abbreviations are used in this manuscript:

RFID	Radio Frequency Identification
GNSS	Global Navigation Satellite System
RSS	Received Signal Strength
WiFi	Wireless Fidelity
PDR	Pedestrian Dead Reckoning
MM	Magnetic Matching
UILoc	Unsupervised Indoor Localization
DL	Deep Learning
CNN	Convolutional Neural Network
AP	Access Point
RSSI	Received Signal Strength Indicator
KNN	K-Nearest Neighbor
SVM	Support Vector Machines
RNN	Recurrent Neural Networks
GRU	Gate Recurrent Units
BLE	Bluetooth Low Energy
RP	Reference Point
EM	Expectation–Maximization
ANOVA	Analysis of Variance
SLAM	Simultaneous Localization and Mapping
TNN	Tensor Nuclear Norm
ASMF	Adaptive signal Mode Fingerprinting
SRL-KNN	Soft Range Limited K-Nearest Neighbor
PDOA	Phase Difference of Arrival
CW	Clockwise
CCW	Counterclockwise
DOSSOM	Dual One Slope with Second Order Model
CDF	Cumulative Distribution Function

## References

[1] Kolomijeca A., López-Salcedo J.A., Lohan E., Seco-Granados G. GNSS applications: Personal safety concerns. Proceedings of the 2016 International Conference on Localization and GNSS (ICL-GNSS).

[2] Hameed A., Ahmed H.A. Survey on indoor positioning applications based on different technologies. Proceedings of the 2018 12th International Conference on Mathematics, Actuarial Science, Computer Science and Statistics (MACS).

[3] Breßler J., Reisdorf P., Obst M., Wanielik G. GNSS positioning in non-line-of-sight context—A survey. Proceedings of the 2016 IEEE 19th International Conference on Intelligent Transportation Systems (ITSC).

[4] Marathe T., Broumandan A., Pirsiavash A., Lachapelle G. Characterization of Range and Time Performance of Indoor GNSS Signals. Proceedings of the 2018 European Navigation Conference (ENC).

[5] Hesslein N., Wesselhöft M., Hinckeldeyn J., Kreutzfeldt J. (2021). Industrial Indoor Localization: Improvement of Logistics Processes Using Location Based Services. Advances in Automotive Production Technology–Theory and Application.

[6] Tsai C., Hsu K. An Application of Using Bluetooth Indoor Positioning, Image Recognition and Augmented Reality. Proceedings of the 2016 IEEE 13th International Conference on e-Business Engineering (ICEBE).

[7] Birla S., Singh G., Kumhar P., Gunjalkar K., Sarode S., Choubey S., Pawar M. Disha-Indoor Navigation App. Proceedings of the 2020 2nd International Conference on Advances in Computing, Communication Control and Networking (ICACCCN).

[8] Fortes S., Aguilar-Garcia A., Barco R., Barba F.B., Fernández-Luque J.A., Fernández-Durán A. (2015). Management architecture for location-aware self-organizing LTE/LTE-A small cell networks. IEEE Commun. Mag..

[9] Fortes S., Baena C., Villegas J., Baena E., Asghar M.Z., Barco R. (2021). Location-Awareness for Failure Management in Cellular Networks: An Integrated Approach. Sensors.

[10] Aguilar-Garcia A., Fortes S., Molina-García M., Sánchez J.C., Alonso J.I., Garrido A., Fernandez-Duran A., Barco R. (2015). Location-aware self-organizing methods in femtocell networks. Comput. Netw..

[11] Aguilar-Garcia A., Fortes S., Fernandez-Duran A., Barco R. (2016). Context-Aware Self-Optimization: Evolution Based on the Use Case of Load Balancing in Small-Cell Networks. IEEE Veh. Technol. Mag..

[12] Zafari F., Gkelias A., Leung K. (2017). A Survey of Indoor Localization Systems and Technologies. arXiv.

[13] Aguilar-Garcia A., Fortes S., Colin E., Barco R. Enhancing localization accuracy with multi-antenna UHF RFID fingerprinting. Proceedings of the 2015 International Conference on Indoor Positioning and Indoor Navigation (IPIN).

[14] Basri C., el Khadimi A. Survey on indoor localization system and recent advances of WIFI fingerprinting technique. Proceedings of the 2016 5th International Conference on Multimedia Computing and Systems (ICMCS).

[15] García-Paterna P.J., Martínez-Sala A.S., Sánchez-Aarnoutse J.C. (2021). Empirical Study of a Room-Level Localization System Based on Bluetooth Low Energy Beacons. Sensors.

[16] Aguilar-Garcia A., Fortes S., Colin E., Barco R. (2015). Enhancing RFID indoor localization with cellular technologies. EURASIP J. Wirel. Commun. Netw..

[17] Ismail H., Kitagawa H., Tasaki R., Terashima K. WiFi RSS fingerprint database construction for mobile robot indoor positioning system. Proceedings of the 2016 IEEE International Conference on Systems, Man, and Cybernetics (SMC).

[18] Seco F., Jimenez A.R. Autocalibration of a wireless positioning network with a FastSLAM algorithm. Proceedings of the International Conference on Indoor Positioning and Indoor Navigation.

[19] Roy P., Chowdhury C. (2021). A Survey of Machine Learning Techniques for Indoor Localization and Navigation Systems. J. Intell. Robot. Syst..

[20] Hatem E., Colin E., Abou-Chakra S., El-Hassan B., Laheurte J.-M. New Empirical Indoor Path Loss Model using Active UHF-RFID Tags for Localization Purposes. Proceedings of the 2018 IEEE International Conference on RFID Technology & Application (RFID-TA).

[21] Ni L.M., Liu Y., Lau Y.C., Patil A.P. LANDMARC: Indoor location sensing using active RFID. Proceedings of the First IEEE International Conference on Pervasive Computing and Communications (PerCom 2003).

[22] Huang Y., Lv S., He Y., Huang J. (2011). An Isosceles Triangular Placement of Reference Tags for RFID Indoor Location System. Chin. J. Electron..

[23] Wu J., Zhu M., Xiao B., Qiu Y. The Improved Fingerprint-Based Indoor Localization with RFID/PDR/MM Technologies. Proceedings of the 2018 IEEE 24th International Conference on Parallel and Distributed Systems (ICPADS).

[24] Chen J., Zhang Y., Xue W. (2018). Unsupervised indoor localization based on Smartphone Sensors, iBeacon and Wi-Fi. Sensors.

[25] Mittal A., Tiku S., Pasricha S. Adapting convolutional neural networks for indoor localization with smart mobile devices. Proceedings of the 2018 on Great Lakes Symposium on VLSI.

[26] Sinha R.S., Hwang S.H. (2019). Sinha Comparison of CNN applications for RSSI-based fingerprint indoor localization. Electronics.

[27] Liu Z., Dai B., Wan X., Li X. (2019). Hybrid wireless fingerprint indoor localization method based on a convolutional neural network. Sensors.

[28] Carvalho E.C., Ferreira B.V., Filho P.R.G., Gomes P.H., Freitas G.M., Vargas P.A., Pessin G. Towards a smart fault tolerant indoor localization system through recurrent neural networks. Proceedings of the 2019 IEEE International Joint Conference on Neural Networks (IJCNN).

[29] Li M., Zhao L., Tan D., Tong X. (2019). BLE fingerprint indoor localization algorithm based on eight-neighborhood template matching. Sensors.

[30] González J.L.S., Morillo L.M.S., Álvarez-García J.A., Ros F.E.D.S., Ruiz A.R.J. (2019). Energy-efficient indoor localization WiFi-fingerprint system: An experimental study. IEEE Access.

[31] Olesiński A., Piotrowski Z. (2021). An Adaptive Energy Saving Algorithm for an RSSI-Based Localization System in Mobile Radio Sensors. Sensors.

[32] Zhao F., Huang T., Wang D. (2019). A probabilistic approach for wifi fingerprint localization in severely dynamic indoor environments. IEEE Access.

[33] Wang W., Marelli D., Fu M. (2020). Fingerprinting-based indoor localization using interpolated preprocessed CSI phases and Bayesian tracking. Sensors.

[34] Thewan T., Ismail A.H., Panya M., Terashima K.T. Assessment of WiFi RSS using design of experiment for mobile robot wireless positioning system. Proceedings of the 2016 19th International Conference on Information Fusion (FUSION).

[35] Nastac D., Lehan E., Iftimie F.A., Arsene O., Cramariuc B. Automatic Data Acquisition with Robots for Indoor Fingerprinting. Proceedings of the 2018 International Conference on Communications (COMM).

[36] Yan H., Peng T., Liu H., Ding Y. Indoor Position Method of Industrial Robot Based on Wifi Fingerprint Position Technology. Proceedings of the 2019 1st International Conference on Industrial Artificial Intelligence (IAI).

[37] Luo R.C., Hsiao T.J. (2018). Dynamic wireless indoor localization incorporating with an autonomous mobile robot based on an adaptive signal model fingerprinting approach. IEEE Trans. Ind. Electron..

[38] Serif T., Perente O.K., Dalan Y. RoboMapper: An Automated Signal Mapping Robot for RSSI Fingerprinting. Proceedings of the 2019 7th International Conference on Future Internet of Things and Cloud (FiCloud).

[39] Trogh J., Joseph W., Martens L., Plets D. (2019). An unsupervised learning technique to optimize radio maps for indoor localization. Sensors.

[40] Sinha R.S., Hwang S.H. (2020). Improved RSSI-based data augmentation technique for fingerprint indoor localization. Electronics.

[41] Hoang M.T., Zhu Y., Yuen B., Reese T., Dong X., Lu T., Xie M. (2018). A soft range limited K-Nearest Neighbors algorithm for indoor localization enhancement. IEEE Sens. J..

[42] Zhang Y., Gong X., Liu K., Zhang S. (2021). Localization and Tracking of an Indoor Autonomous Vehicle Based on the Phase Difference of Passive UHF RFID Signals. Sensors.

[43] Loganathan A., Ahmad N.S., Goh P. (2019). Self-adaptive filtering approach for improved indoor localization of a mobile node with zigbee-based RSSI and odometry. Sensors.

[44] Adept MobileRobots (2011). Pioneer 3-DX Datasheet. http://www.mobilerobots.com/Libraries/Downloads/Pioneer3DX-P3DX-RevA.sflb.ashx.

[45] Park S., Hashimoto S. (2009). Autonomous Mobile Robot Navigation Using Passive RFID in Indoor Environment. IEEE Trans. Ind. Electron..

[46] Borenstein J., Feng L. (1994). UMBmark—A Method for Measuring, Comparing, and Correcting Dead-Reckoning Errors in Mobile Robots.

[47] Sadowski S., Spachos P. (2018). Rssi-based indoor localization with the internet of things. IEEE Access.

[48] Ela Innovation S.A. Ela Innovation Active RFID Tag and Reader Manufacturer. https://elainnovation.com/.

[49] Fornaser A., Maule L., Luchetti A., Bosetti P., de Cecco M. (2019). Self-Weighted Multilateration for Indoor Positioning Systems. Sensors.

